# Correspondence: Chimpanzee helping is real, not a byproduct

**DOI:** 10.1038/s41467-017-02321-6

**Published:** 2018-02-12

**Authors:** Alicia P. Melis, Jan M. Engelmann, Felix Warneken

**Affiliations:** 1 Warwick Business School, The University of Warwick, Coventry, CV4 7AL UK; 2 Department of Developmental and Comparative Psychology, Max Planck Institute for Evolutionary Anthropology, Leipzig, 04103 Germany; 3 Department of Psychology, University of Michigan, Ann Arbor, MI 48109-1043 USA

## Introduction

In their recent study, Tennie et al.^1^ argue that positive instances of chimpanzees helping others can be a byproduct of testing methods^1^. The study includes a new task where chimpanzees can behave prosocially toward a conspecific either through an action (GO-condition) or by omission (NO-GO condition). The study further aims to test whether stimulus enhancement or carry-over effects from prior experiences explain previous results. We agree that a helping-by-omission task could in principle provide intriguing new evidence for chimpanzee helping. However, here we raise a number of crucial methodological issues that question the current interpretation of the study’s results. Furthermore, the study fails to consider the evidence from prior work addressing these concerns.

First, in any study on animal prosociality, showing apparatus understanding is key. However, it is unclear whether chimpanzees understood critical aspects of the GO/NO-GO task used. One group of chimpanzees had access to an apparatus where releasing a peg provided access to food from a box (GO condition), while in another group releasing the peg blocked the box (NO-GO). However, chimpanzees failed a post-test designed to test apparatus understanding. Therefore, the only conclusion that can be drawn from Experiment 1 is that the physical causality of these apparatuses was too complicated for the chimpanzees to understand through observation, not anything about helping.

This lack of comprehension also undermines the conclusions from Experiment 2 where chimpanzees could learn about the apparatuses prior to the test phase. A closer look at the methods reveals that the knowledge probe from the NO-GO task does not actually demonstrate comprehension. This is because subjects were introduced to the NO-GO apparatus when it was already delivering peanuts in Steps 1 and 2 of the familiarization, so chimpanzees could ignore the peg, start feeding, and not learn the relevance of the peg blocking or unblocking the apparatus. Therefore, not releasing the peg does not provide clear evidence for apparatus understanding. A better test would have been to attach the peg before moving to the food-delivery room. This would have provided convincing evidence for subjects’ understanding of the instrumental relationship between the presence of the peg and the possibility to obtain food. Given the lack of evidence about chimpanzees’ comprehension of the NO-GO helping-my omission condition, the only validated data come from the action-version of the task (GO condition compared with a social control). This shrinks the usable sample to *n* = 6 participants, severely weakening the conclusions.

Second, it is unclear whether the need for help was obvious to potential donors. This is critical because signaling a need for help is the primary factor predicting whether chimpanzees help in all prior helping studies^2–6^. In fact, one theory is that chimpanzees engage in reactive prosocial behavior when recipients signal their need, but do not help proactively in the absence of overt cues^3,7^. We performed a meta-analysis to investigate whether signaling need predicted chimpanzees’ helping across six previous published studies (Table 1). The analysis revealed a strong effect of chimpanzees’ signaling of need on helping behavior (Cohen’s *d* = 0.73). It is unclear whether the chimpanzees would have been able to detect any signals of need in the setup used by Tennie et al.^1^. The recipients manipulated a box that, depending on condition, either did or did not dispense peanuts. Yet because the peanuts fell into an opaque trough away from the potential helper, it is unclear whether the helper even knew whether the recipient obtained food. The recipient’s need for help is therefore much less salient and clear than in prior work where recipients directly reached for inaccessible objects, failed to open a locked door, or tried to access a bag of food in full sight of the subject.

**Table 1:** Studies included in the meta-analysis assessing signaling need and helping

Study	*N*	*d*	Signaling behavior	Population
Warneken and Tomasello^4^	3	1.51	Reaching for object	WKPRC, Zoo Leipzig
Warneken et al.^2^, Study 1	36	0.93	Reaching for object	Ngamba Island Chimpanzee Sanctuary
Warneken et al.^2^, Study 2	18	0.36	Reaching for object	Ngamba Island Chimpanzee Sanctuary
Warneken et al.^2^, Study 3	9	0.76	Trying to open door	Ngamba Island Chimpanzee Sanctuary
Yamamoto et al.^5^, Study 1^c^	9	0.46^b^	Reaching for tool	Primate Research Institute, Kyoto University
Melis et al.^3^	14	0.72	Manipulating apparatus, attention-getters	Ngamba Island Chimpanzee Sanctuary

^a^We used original data for all studies to calculate Cohen’s *d*. We then calculated a weighted mean estimate of the effect size for each study. This was done in order to give more emphasis to results obtained from larger samples. To do so, we weighted each study’s Cohen’s *d* by its respective sample size. We multiplied the *N* by the *d* for each study and then summed the results. This result was then divided by the combined sample size of all studies

^b^This is based on the authors’ analysis of tool transfers with and without request

^c^We could not include ref. ^5^ (Study 2) and ref. ^6^ because the authors' analyses focused on trials with helping only and assessed what proportion of these helping trials were preceded by signals from the recipient. This is the reversal of the other studies where signaling behavior was used as an independent variable to assess whether it results in more or less helping as a dependent variable

Third, the study provides evidence contradicting the stimulus-enhancement hypothesis, contrary to the study’s conclusion. This hypothesis states that chimpanzees provide help to conspecifics not in order to benefit others, but simply because their attention is drawn to the helping apparatus as a result of the recipient’s actions. In this view, helping is not prosocially motivated, but amounts to what the authors call ‘by-product helping’. In the current setup, the stimulus-enhancement hypothesis makes two key predictions: (1) there should be no difference in rates of peg releases for the GO and NO-GO groups, because subjects’ attention is drawn to the target action equally in both conditions and (2) peg release rates should differ between the test and social control conditions, for both the GO and NO-GO groups, because potential recipients are off to the side in the social control condition and cannot draw subjects’ attention to the food apparatus. Yet, critically, the present setup does not allow for a conclusive test of the first prediction given that there is no evidence that the NO-GO group understood the apparatus, as described above. The second prediction was tested, but is not supported by the actual results.: Across both the GO and NO-GO subjects, chimpanzees do not show increased release rates in the test condition compared to the social control condition. Therefore, the results are not consistent with the stimulus-enhancement hypothesis.

**Fig. 1:**
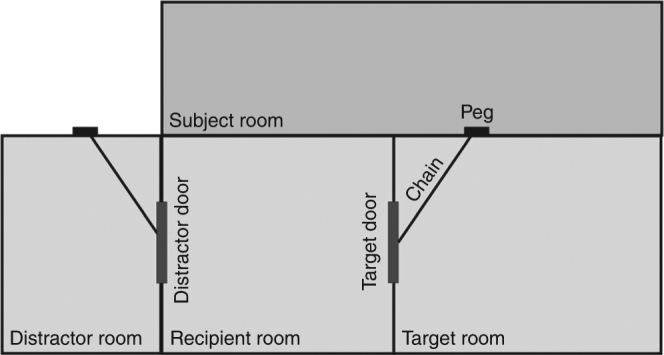
Testing setup from Warneken et al.^2^, Experiment 3. Both the target and the distractor door were held shut by chains. In the Experimental condition, food was placed in the target room, so that the recipient would try to open the target door and the subject could help by releasing the chain blocking the target door from another room. In the Control condition, food was placed in the distractor room, so that the recipient would try to open the distractor door. Results showed that subjects released the chain significantly more often in the Experimental than the Control condition

A direct test of the stimulus-enhancement hypothesis would require testing if enhancing the stimulus also leads to more target behavior. We assessed this for a study in which it was found that chimpanzees release a chain to unlock a door for a conspecific when he tries to open the door (but not when he ignores the door, Fig. 1)^2^. We found that the movement of the chain itself was not predictive of helping behavior, and in fact tended to be negatively related to helping (*r*_Spearman_ (*n* = 37) = −0.293, *p* = 0.079, Supplementary Methods). The increased chain movement was likely the result of the recipient’s more forceful attempts to open the door when no help came forward, rather than a cause for the chimpanzee subject to release it.

Further evidence against the stimulus-enhancement hypothesis comes from several published studies. First, multiple experiments have used situations in which the target objects were simply out of the recipient’s reach, such that the recipient could not physically manipulate them at all^2,4–6^. Although this removes any possibility of stimulus enhancement, chimpanzees still helped more in test conditions than control conditions. Second, another study examined how chimpanzees responded to a conspecific exhibiting an ambiguous reach in the direction of two potential tools^6^. Subjects reliably provided the tool the recipient actually needed in their given situation, inferring the best way to help based on context—which cannot be explained by stimulus-enhancement accounts. Third, it has been found that chimpanzees help those individuals more who have helped them previously^8–12^. If stimulus enhancement were to account for helping, no such difference based upon the prior social history should occur. None of this evidence is considered in the current study.

In conclusion, converging evidence shows that chimpanzees are willing to help others by doing something^7,13^; whether chimpanzees also help by doing nothing is an interesting question for future research.

## Methods

For the meta-analysis, we calculated Cohen’s *d* from all studies that had assessed the occurrence of recipient cues and its effect on helping. We then calculated a weighted mean estimate of the effect size for each study to account for sample size.

A previous study^2^ showed that chimpanzees unleashed a chain when it blocked a door that another chimpanzee tried to open in an Experimental condition (vs. a Control where the door was ignored). We coded the degree of chain movement of the Experimental condition on a 5-point scale, resulting in high inter-rater agreement (weighted *κ* = 0.86, see Supplementary Methods).

We coded the degree of chain movement of the Experimental condition from Warneken et al. (2007, Study 3), from a video, on a 5-point scale. From the total of 45 test trials, 37 trials could be included in this analysis. Five additional trials were missing due to problems with video recording and three trials could not be coded with our coding schema for chain movement because subject directly manipulated the chain. A second coder independently rated 50% of events blind to hypotheses and condition. Inter-rater agreement was high, Cohen’s weighted *κ* (quadratic) = 0.86.

## Electronic supplementary material

Supplementary Information

## References

[1] Tennie, C., Jensen, K. & Call, J. The nature of prosociality in chimpanzees. *Nat. Commun.* 7, 10.1038/ncomms13915 (2016). 10.1038/ncomms13915 PMC5187495 27996969

[2] Warneken F, Hare B, Melis AP, Hanus D, Tomasello M (2007). Spontaneous altruism by chimpanzees and young children. PLoS Biol..

[3] Melis AP (2011). Chimpanzees help conspecifics obtain food and non-food items. Proc. Biol. Sci..

[4] Warneken F, Tomasello M (2006). Altruistic helping in human infants and young chimpanzees. Science.

[5] Yamamoto S, Humle T, Tanaka M (2009). Chimpanzees help each other upon request. PLoS ONE.

[6] Yamamoto S, Humle T, Tanaka M (2012). Chimpanzees’ flexible targeted helping based on an understanding of conspecifics’ goals. Proc. Natl Acad. Sci. USA.

[7] Melis AP, Warneken F (2016). The psychology of cooperation: insights from chimpanzees and children. Evolut. Anthropol..

[8] Melis AP, Hare B, Tomasello M (2008). Do chimpanzees reciprocate received favours?. Anim. Behav..

[9] Engelmann JM, Herrmann E, Tomasello M (2015). Chimpanzees trust conspecifics to engage in low-cost reciprocity. Proc. Biol. Sci..

[10] Muller MN, Mitani JC (2005). Conflict and cooperation in wild chimpanzees. Adv. Study Behav..

[11] Gomes CM, Mundry R, Boesch C (2009). Long-term reciprocation of grooming in wild West African chimpanzees. Proc. Biol. Sci..

[12] Schmelz M, Grueneisen S, Kabalak A, Jost J, Tomasello M (2017). Chimpanzees return favors at a personal cost. Proc. Natl Acad. Sci. USA.

[13] de Waal F (2008). Putting the altruism back into altruism: the evolution of empathy. Annu. Rev. Psychol..

