# Supplementary material for: Correspondence: Chimpanzee helping is real, not a byproduct
Source: Nat Commun. 2018 Feb 12;9:615. doi: 10.1038/s41467-017-02321-6 (PMC5809601; doi:10.1038/s41467-017-02321-6)
Supplement: Supplementary file 1 — Supplementary Information [file 41467_2017_2321_MOESM1_ESM.pdf]

Supplementary Methods

We coded the degree of chain movement of the Experimental condition from Warneken et al. (2007, Study 3) from video on a 5-point scale. From the total of 45 test trials, 37 trials could be included in this analysis. Five additional trials were missing due to problems with video recording and three trials could not be coded with our coding schema for chain movement because subject directly manipulated the chain. A second coder independently rated 50% of events blind to hypotheses and condition. Interrater agreement was high, Cohen's weighted kappa (quadratic) = .86.
